# Management of complicated choledochal cyst in children: ultrasound-guided percutaneous external drainage and subsequent definitive operation

**DOI:** 10.1186/s12887-023-03994-3

**Published:** 2023-05-26

**Authors:** Jiayu Yan, Chuankai Lv, Dan Zhang, Mingkang Zheng, Chunhui Peng, Wenbo Pang, Wei Chen, Siwei Wang, Xiaoman Wang, Yajun Chen

**Affiliations:** 1 Department of General Surgery, Beijing Children’s Hospital, Capital Medical University, National Center for Children’s Health, Beijing, 100045 China; 2 Department of Ultrasound, Beijing Children’s Hospital, Capital Medical University, National Center for Children’s Health, No. 56 Nanlishi Road, Xicheng District, Beijing, 100045 China; 3 Department of Surgery, Zhuhai City Maternity and Child Health Hospital, Zhuhai, Guangdong Province 519001 China

**Keywords:** Choledochal cyst, Ultrasound, Percutaneous external drainage, Definitive operation

## Abstract

**Objective:**

The purpose of this study was to analyze the outcomes of the combination of ultrasound (US)-guided percutaneous external drainage and subsequent definitive operation to manage complicated choledochal cyst in children.

**Methods:**

This retrospective study included 6 children with choledochal cyst who underwent initial US-guided percutaneous external drainage and subsequent cyst excision with Roux-en-Y hepaticojejunostomy between January 2021 and September 2022. Patient characteristics, laboratory findings, imaging data, treatment details, and postoperative outcomes were evaluated.

**Results:**

Mean age at presentation was 2.7 ± 2.2 (0.5–6.2) years, and 2 patients (2/6) were boys. Four patients (4/6) had a giant choledochal cyst with the widest diameter of ≥ 10 cm and underwent US-guided percutaneous biliary drainage on admission or after conservative treatments. The other 2 patients (2/6) underwent US-guided percutaneous transhepatic cholangio-drainage and percutaneous transhepatic gallbladder drainage due to coagulopathy, respectively. Five patients (5/6) recovered well after US-guided percutaneous external drainage and underwent the definitive operation, whereas 1 patient (1/6) had liver fibrosis confirmed by Fibroscan and ultimately underwent liver transplantation 2 months after external drainage. The mean time from US-guided percutaneous external drainage to the definitive operation was 12 ± 9 (3–21) days. The average length of hospital stay was 24 ± 9 (16–31) days. No related complications of US-guided percutaneous external drainage occurred during hospitalization. At 10.2 ± 6.8 (1.0–18.0) months follow-up, all patients had a normal liver function and US examination.

**Conclusions:**

Our detailed analysis of this small cohort suggests that US-guided percutaneous external drainage is technically feasible for choledochal cyst with giant cysts or coagulopathy in children, which may provide suitable conditions for subsequent definitive operation with a good prognosis.

**Trial registration:**

Retrospectively registered.

## Background

Choledochal cyst, a relatively uncommon congenital malformation characterized by biliary tract dilatation with an incidence of around 1:13,000 in Asian populations versus 1:100,000 in Western populations, often presents with abdominal pain, abdominal lump, and jaundice in pediatric patients [1–3]. The currently acknowledged etiology of choledochal cyst is the anomalous pancreaticobiliary junction with reflux of pancreatic juice into the common bile duct and the formation of protein plugs or calculi in the bile duct [4, 5]. Given that patients with choledochal cyst have a high risk of hepatobiliary complications, such as cholangitis, pancreatitis, perforation of the cyst, and potential malignant change, prompt treatment is required [1, 6, 7]. Some studies have reported that even if patients are asymptomatic, surgical intervention as soon as possible can lead to a better outcome, such as a significantly reduced rate of liver fibrosis in patients [3, 8]. The specific approach is largely a function of the type of cyst and the preferred treatment is total cyst excision with Roux-en-Y hepaticojejunostomy [8, 9]. However, surgical intervention should be elective, some related concomitant complications, including cholangitis, pancreatitis, and coagulopathy, may require conservative treatments, delay the surgical treatments, and become the major risk for operation [10–12]. How to address complicated choledochal cyst has always been challenging in clinical management.

**Fig. 1:**
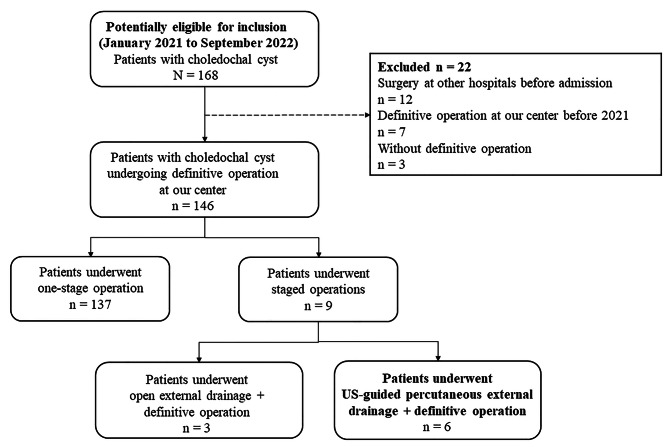
Flow diagram of the study population

Patients with complicated choledochal cyst often had a distal biliary obstruction that led to bile stasis and ultimately developed severe complications, such as acute cholangitis, abnormal liver function, and even perforation of choledochal cysts and coagulopathy [11–13]. These patients commonly required two-stage operations, urgent external biliary drainage, and subsequent definitive operation [12, 14, 15]. The methods of external drainage mainly included biliary drainage of extrahepatic cyst or intrahepatic bile duct, and gallbladder drainage (cholecystostomy) [11, 15–17]. In most pediatric cases, traditional external drainage was accomplished by laparotomy or laparoscopic-assisted [11, 12, 18]. Percutaneous external drainage of choledochal cyst in children has been rarely reported in the literature, and ultrasound (US)-guided percutaneous external drainage is even rarer [11, 15].

**Fig. 2:**
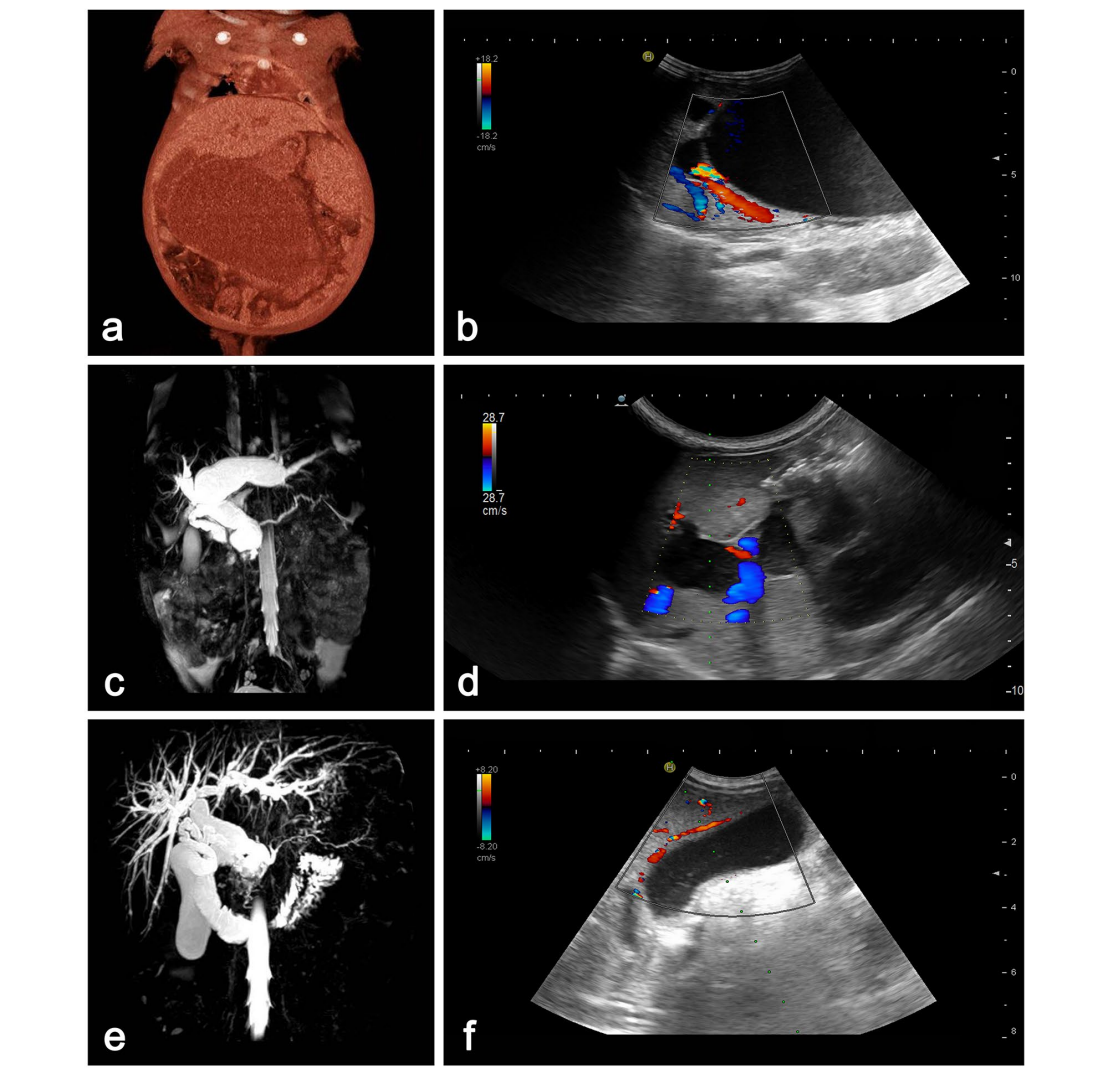
CT or MRCP, and US results of patients with choledochal cyst before and during US-guided percutaneous external drainage. (**A**) CT before US-guided PBD, Case 1 with a giant choledochal cyst. (**B**) US-guided PBD, Case 1 with a giant choledochal cyst. (**C**) MRCP before US-guided PTCD, Case 5 with significant dilatation of the intrahepatic bile duct. (**D**) US-guided PTCD, Case 5 with significant dilatation of the intrahepatic bile duct. (**E**) MRCP before US-guided PTGD, Case 6 with a large gallbladder. (**F**) US-guided PTGD, Case 6 with a large gallbladder

This study aimed to report the early clinical experience of the combination of US-guided percutaneous external drainage and subsequent definitive operation to manage choledochal cyst in children in a small cohort and evaluate its safety and effect.

## Methods

### Patients

This retrospective study was approved by the Ethics Committee of Beijing Children’s Hospital (Approval number: 2022-E-214-R) and the need for informed consent was waived. The medical records of patients diagnosed with choledochal cyst and admitted to the department of general surgery, Beijing Children’s Hospital, from January 2021 to September 2022, were reviewed. The diagnosis of choledochal cyst was based on abdominal US with computed tomography (CT) or magnetic resonance cholangiopancreatography (MRCP). Patients who underwent US-guided percutaneous external drainage and subsequent definitive operation (cyst excision with Roux-en-Y hepaticojejunostomy) were included in this study. The exclusion criteria were as follows: (I) underwent surgery at other hospitals before admission; (II) underwent the definitive operation at our center before 2021; (III) no definitive operation; (IV) underwent one-stage definitive operation; and (V) underwent traditional open external drainage and definitive operation (Fig. 1).

**Fig. 3:**
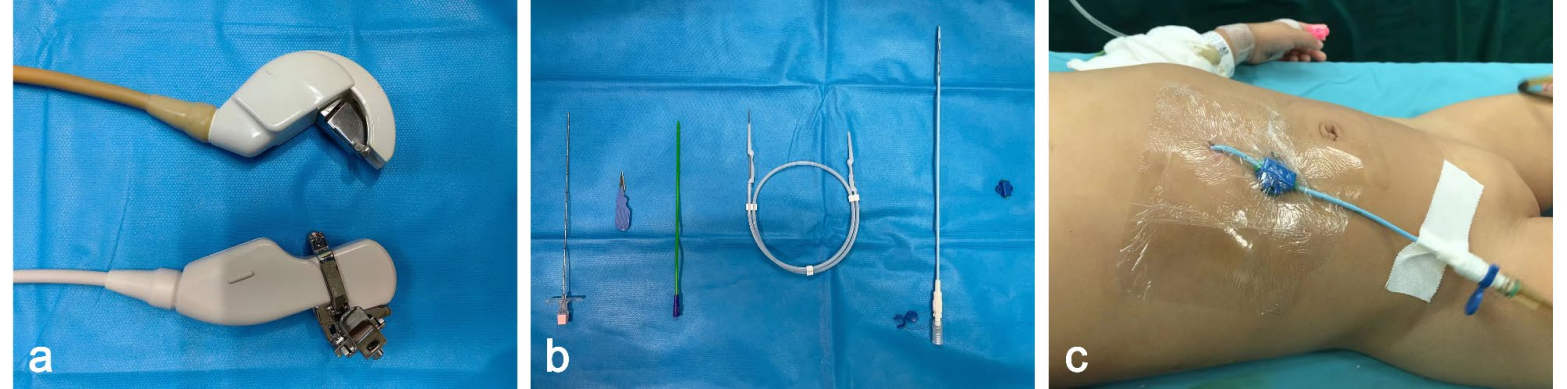
Operating instruments in US-guided percutaneous external drainage. (**A**) Ultrasound probes. (**B**) Mini-invasive kits of puncture, including puncture needle, sharp knife, tract dilator, guide wire, Pig-tail catheter, and external fixator. (**C**) The method of external fixation

**Table 1:** Patients and characteristics

Cases	Sex	Age (years)	Types	Size (cm^3^)	Preoperative complications	Main indication for external drainage	Procedures	Definitive procedures^*^	Follow-up (months)
1	M	1.4	IVa	15.2*12.6*9.4	Coagulopathy, abnormal liver function, liver fibrosis	Giant cyst, coagulopathy	US-guided PBD, local anesthesia	Liver transplantation	18.0
2	F	0.5	Ia	20.0*11.2*8.2	Abnormal liver function	Giant cyst	US-guided PBD, general anesthesia	Open	16.0
3	F	6.2	Ia	13.1*5.3*4.9	Abnormal liver function	Giant cyst	US-guided PBD, general anesthesia	Robotic	8.5
4	M	0.9	IVa	10.5*6.6*4.7	Coagulopathy	Giant cyst, coagulopathy	US-guided PBD, general anesthesia	laparoscopic	4.1
5	F	3.2	IVa	5.0*2.1*2.1	Coagulopathy, abnormal liver function, acute pancreatitis	Coagulopathy	US-guided PTCD, general anesthesia	laparoscopic	13.6
6	F	3.8	Ic	1.3^**^	Coagulopathy, abnormal liver function, acute pancreatitis	Coagulopathy	US-guided PTGD, local anesthesia	laparoscopic	1.0

M: Male; F: Female; US: Ultrasound; PBD: Percutaneous biliary drainage; PTCD: Percutaneous transhepatic cholangio-drainage; PTGD: Percutaneous transhepatic gallbladder drainage

^*^ Except for liver transplantation, the procedure performed for patients with choledochal cyst in our department was cyst excision with Roux-en-Y hepaticojejunostomy ^**^ The maximum diameter of the cyst in Ic type of choledochal cyst

**Fig. 4:**
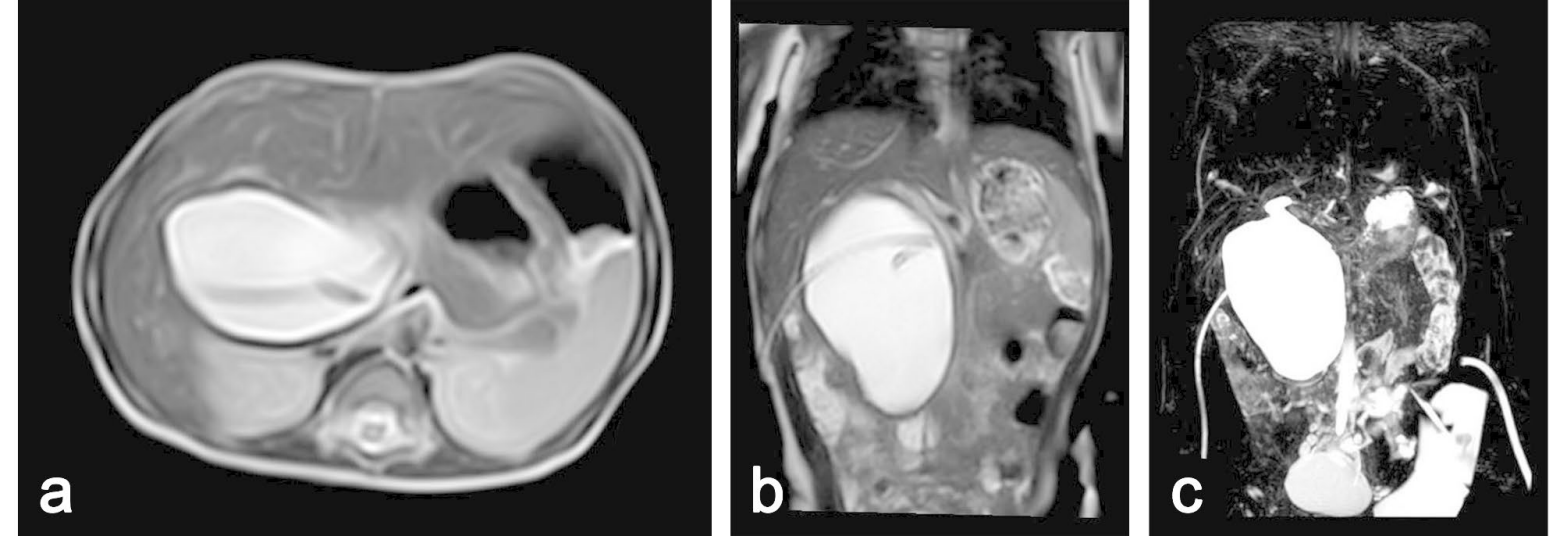
Images of biliary drain after US-guided PBD. (**A**) Axial T2-weighted MR image after US-guided PBD, Case 2. (**B**) Coronal T2-weighted MR image after US-guided PBD, Case 2. (**C**) MRCP image after US-guided PBD, Case 2

### Clinical characteristics

Two pediatric surgeons reviewed the electronic medical records (JYY and MKZ). Patient characteristics, laboratory findings, imaging data, treatment details, and postoperative outcomes were analyzed. Patient characteristics included sex, age, presenting symptoms, features of the choledochal cyst (types and size), and preoperative hepatobiliary complications. The types of choledochal cyst were defined using Todani’ s classification, and the choledochal cyst with a maximum diameter of ≥ 10 cm was defined as a giant cyst [19, 20]. The laboratory findings included the laboratory results before US-guided percutaneous external drainage and before the definitive operation, covering blood routine, coagulation biomarkers, liver function tests and indicators of pancreatitis. The normal reference intervals for laboratory results in our study were based on the PRINCE study [21]. Fibrinogen (FIB) level < 2 g/L with prolonged prothrombin time (PT) or activated partial thromboplastin time (APTT) indicated coagulopathy [12, 22]. The value of aspartate aminotransferase (AST) and alanine aminotransferase (ALT) > 45 U/L with γ-glutamyl transpeptidase (γ-GGT) > 70 U/L indicated abnormal liver function [23]. Acute pancreatitis was characterized by abdominal pain and elevated serum amylase (AMY) and lipase (LPS) levels (> 2 times the upper limit of reference interval) [24]. In addition, for patients with features of liver parenchymal disease indicated by US, the degree of liver fibrosis would be evaluated by Fibroscan, and the value of liver stiffness measurement > 15.15 kPa indicated liver fibrosis in patients [20, 25].

**Fig. 5:**
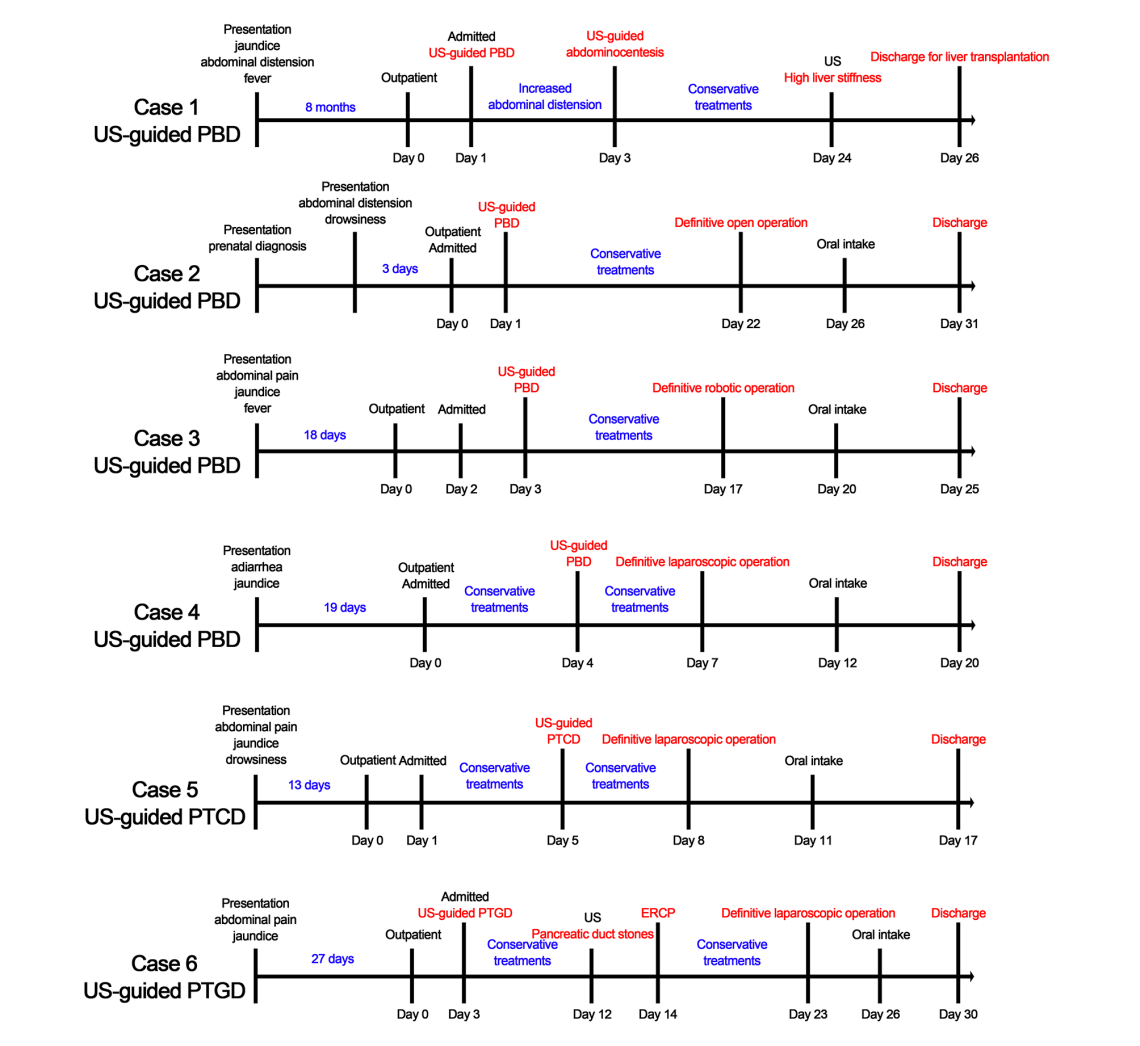
Clinical diagnosis and treatment timelines of patients with choledochal cyst who underwent US-guided percutaneous external drainage and subsequent definitive operation

The imaging data included the images of US, CT, and MRCP performed at our center, including images during US-guided percutaneous external drainage. The treatment details had three aspects: conservative, interventional, and surgical treatments. The conservative treatments of each patient were based on the laboratory results on admission, mainly including nil-per-os, somatostatin injected with a microinfusion pump (3.5 µg/kg/h), intravenous omeprazole (1–2 mg/kg), antibiotic treatment, and total parenteral nutrition. Telephone interviews were performed in October 2022 to ask the patients’ parents about the child’s prognosis and postoperative complications.

### Ultrasound-guided percutaneous external drainage

The decision to perform external drainage was based on the patients’ laboratory and imaging results (CT or MRCP). If a patient had a giant cyst with abnormal liver function or especially coagulopathy and did not improve after conservative treatments, US-guided percutaneous external drainage would be considered. Every patient fasted for a minimum of 6 h and was re-evaluated by US to determine the extent of intrahepatic bile duct dilation, the size of extrahepatic cyst and gallbladder to help select the specific methods of external drainage. The US-guided percutaneous external drainage was performed by a pediatric radiologist (CKL) with more than 5 years of experience in interventional US techniques, after obtaining informed consent from the parents of patients. The preferred method was US-guided percutaneous biliary drainage (PBD), especially for patients with a giant extrahepatic cyst. In addition, US-guided percutaneous transhepatic cholangio-drainage (PTCD) was performed in patients with a small extrahepatic cyst, but significant dilatation of the intrahepatic bile duct, and US-guided percutaneous transhepatic gallbladder drainage (PTGD) was performed in patients with a large gallbladder (Fig. 2).

All procedures are accomplished under general anesthesia by anesthesiologists or local anesthesia with the support of oral chloral hydrate as a sedating agent by continuous close monitoring, including the preparation of emergency medications and oxygen therapy devices. At the supine position, the right upper quadrant of patients was draped in a sterile manner. Lidocaine (2%) was used as a local anesthetic. First, after the puncture point was determined by ultrasound and the skin was cut through by a sharp scalpel, the right position of the puncture needle was ensured by real-time US guidance and introduced guide wire. This process should be far away from the abdominal organs and blood vessels as possible. Second, after the puncture, tract dilation was done under real-time US guidance. Finally, a Pig-tail catheter (6–8 Fr) was inserted along the introduced guide wire to decompress the biliary system, and the external fixation to the skin was made (Fig. 3). In US-guided PBD, the point at the anterior axillary line with the smallest distance between the cyst and abdominal wall was selected as the puncture point. In US-guided PTCD and US-guided PTGD, the puncture needle was passed through the liver to penetrate the target intrahepatic bile duct or gallbladder without touching the intrahepatic blood vessels.

After external drainage, conservative treatments were performed, including intravenous broad-spectrum antibiotics and hemostatic agents, and patients resumed oral intake as soon as possible to avoid electrolyte disturbances. Laboratory tests were performed on the 1st, 3rd, and 7th day after external drainage to check the improvement of the liver and coagulation function. Once needed, patients were re-examined every 7 days until the liver and coagulation function was appropriate for definite surgery. Usually in our center, CT or MRCP would be repeated prior to the definitive operation (Fig. 4).

**Table 2:** Results of laboratory tests in 6 patients^*^

Case	CRP (mg/L)	WBC (10^9^/L)	FIB (g/L)	PT (S)	APTT (S)	NH_3_ (umol/L)	AST (U/L)	ALT (U/L)	γ-GGT (U/L)	DBIL (umol/L)	IBIL (umol/L)	AMY (U/L)	LPS (U/L)
Reference interval	<10.0	4.4–11.9	2.0–4.0	9.4–2.5	25.1–38.4	18.0–72.0	14.0–44.0	7.0–30.0	5.0–19.0	0.0-3.42	0.0-17.1	0.0-125.0	0.0–39.0
Case 1													
Before external drainage	**15.0**	9.9	**1.9**	**17.9**	34.8	**115.0**	**250.6**	**141.7**	**245.8**	**144.7**	**49.6**	81.0	**144.4**
Pre-operation^**^	<10.0	5.5	**1.7**	**17.0**	37.1	**80.0**	**181.4**	**83.0**	**90.4**	**45.1**	**25.6**	11.0	6.0
Case 2													
Before external drainage	<10.0	11.0	3.3	**18.7**	34.7	——	**354.5**	**278.1**	**1751.9**	**40.1**	12.4	31.0	**206.8**
Pre-operation	<10.0	7.16	——	——	——	——	40.1	27.2	**336.2**	2.8	9.6	——	——
Case 3													
Before external drainage	<10.0	9.0	3.4	9.2	35.2	——	**557.0**	**615.9**	**1542.0**	**55.0**	**17.3**	74.0	——
Pre-operation	<10.0	9.7	——	——	——	——	**132.0**	**133.0**	**265.2**	**9.7**	15.3	106	——
Case 4													
Before external drainage	<10.0	**18.4**	**1.9**	**117.4**	**172.0**	32.0	**93.1**	**42.2**	**60.2**	**100.8**	**35.9**	——	——
Pre-operation	<10.0	9.0	**1.9**	11.2	**45.0**	——	**75.7**	23.9	**28.5**	**103.9**	**43.0**	——	——
Case 5													
Before external drainage	<10.0	9.4	**0.3**	**21.5**	32.2	68.0	**254.0**	**284.7**	**748.9**	**96.0**	**38.2**	**1330.0**	**382.5**
Pre-operation	<10.0	5.7	2.0	**14.8**	35	**83.0**	33.9	**52.7**	**362.1**	**9.2**	**19.5**	115.0	27.5
Case 6													
Before external drainage	<10.0	11.1	**1.7**	**30.7**	**45.6**	——	**265.4**	**210.2**	**597.4**	**133.8**	**55.3**	**365.0**	**1929.2**
Pre-operation	<10.0	6.86	2.1	10.9	36.0	——	**60.7**	**33.3**	**74.2**	**5.9**	**21.0**	57.0	30.5

CRP: C-reactive protein; WBC: White blood cell; FIB: Fibrinogen; PT: Prothrombin time; APTT: Activated partial thromboplastin time; NH_3_: Ammonia; AST: Aspartate aminotransferase; ALT: Alanine aminotransferase; γ-GGT: γ-glutamyl transpeptidase; DBIL: Direct bilirubin; IBIL: Indirect bilirubin; AMY: Amylase; LPS: Lipase

^*^ Numbers in bold indicated higher than the upper limit of reference interval

^**^ The laboratory results re-examined in 21 days after ultrasound-guided percutaneous external drainage

### Data analysis

Continuous variables were expressed as the mean and SD with ranges. Categorical variables were expressed as numbers and proportions.

## Results

During the study period, 6 patients with choledochal cyst were treated by a combination of US-guided percutaneous external drainage and subsequent definitive operation, and their clinical characteristics were provided in Table 1. The mean age at presentation was 2.7 ± 2.2 (0.5–6.2) years, and 2 patients (2/6) were boys. Their most common presenting symptoms were jaundice (5/6) and abdominal pain (3/6). Except for case 1, other patients visited our center within 1 month of presenting symptoms (Fig. 5).

**Fig. 6:**
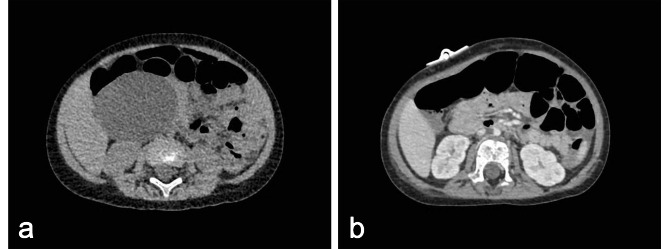
The size of choledochal cyst before and after US-guided percutaneous external drainage. (**A**) CT before US-guided PBD, Case 4. (**B**) CT after US-guided PBD, Case 4

According to the laboratory and imaging results, the main indications for US-guided percutaneous external drainage were giant cyst and coagulopathy. Four patients (4/6, Cases 1–4) had a giant choledochal cyst and underwent US-guided PBD on admission or after conservative treatments. The size of their cysts decreased after US-guided PBD (Fig. 6). The other 2 patients (2/6, Cases 5 and 6) underwent US-guided PTCD and US-guided PTGD due to coagulopathy, respectively (Table 2). Of these patients, 4 patients (4/6, Cases 2–5) underwent external drainage under general anesthesia, and another 2 (2/6, Cases 1 and 4) under local anesthesia. Five patients (5/6) recovered well after external drainage and subsequently underwent the definitive operation, whereas 1 patient (1/6, Case 1) was considered to have liver fibrosis with the value of liver stiffness measurement of 58.8 kPa by Fibroscan 21 days after external drainage and ultimately underwent liver transplantation in another hospital 2 months after external drainage. The mean time from US-guided percutaneous external drainage to the definitive operation was 12 ± 9 (3–21) days. The average length of hospital stay was 24 ± 9 (16–31) days. No infection, electrolyte disturbance, and other complications of US-guided percutaneous external drainage occurred during hospitalization. At 10.2 ± 6.8 (1.0–18.0) months follow-up, all patients had a normal liver function and US examination.

## Discussion

The study demonstrated the feasibility of the combination of US-guided percutaneous external drainage and subsequent cyst excision with Roux-en-Y hepaticojejunostomy to manage the complicated choledochal cyst in children, especially for choledochal cyst with giant cysts or coagulopathy, although the applicable conditions should be further studied given the small sample size of patients.

Early cyst excision has been recommended for children with choledochal cyst to reduce the risk of long-term complications, such as biliary infections and cancer development [26–28]. Moreover, with the improvement of perioperative management and surgical techniques, most patients can undergo one-stage definitive operation [29, 30]. However, in some cases, severe common bile duct (CBD) dilatation, liver dysfunction, or coagulopathy due to biliary obstruction still leads to high surgical risk [12, 18]. Severe CBD dilatation often suggests the presence of CBD stenosis. It can be accompanied by bile duct inflammation, which will lead to repeated infection before the definitive operation, increase the difficulty of surgical operations, and have a high potential for anastomotic leakage during the primary closure [29, 31]. Liver dysfunction and coagulopathy also have a higher risk of anesthesia, intraoperative bleeding, and postoperative disseminated intravascular coagulation. Therefore, timely external drainage is still essential for complicated choledochal cyst [10, 11].

US is a good method to diagnose biliary diseases, including choledochal cyst, and US-guided percutaneous external drainage has been widely used in adult biliary disorders, such as acute cholecystitis, but few studies have reported its application in pediatric patients [8, 32]. Compared with fluoroscopy-guided external drainage, US-guided percutaneous external drainage can avoid unnecessary radiation exposure and reduce vascular injury during operation to prevent bleeding [15, 16]. However, visualization of the bile duct cannot be fully realized by US, so we suggest that cholangiography, CT, or MRCP should be performed consequently before the definitive operation to understand the morphologic features of the whole biliary tract and to check the location of the drainage catheter [16]. In addition, in this small cohort study, we found that patients could undergo appropriate external drainage (PBD, PTCD or PTGD) according to the extent of intrahepatic bile duct dilation, the size of extrahepatic cyst and gallbladder re-evaluated by US before external drainage, which had not been reported in previous studies. However, US is a very user-dependent imaging modality, and proper skills and rich experience of the radiologists are required to ensure the safety of patients, which is why US-guided percutaneous external drainage is not widely used in pediatric patients [33].

For patients with complicated choledochal cyst, US-guided percutaneous external drainage is minimally invasive and effective in relieving the symptoms of biliary obstruction, but it also has risks of electrolyte disturbance and infection [11, 16, 18]. It has been reported that the coagulation function of patients with choledochal cyst improved significantly 7 days after external drainage [12]. Cases 4–6 in our study with coagulopathy on admission were improved after US-guided percutaneous external drainage. To reduce the associated complications of US-guided percutaneous external drainage, all patients underwent the definitive operation within 21 days after external drainage during one hospitalization. If the liver and coagulation function of patients cannot be improved after US-guided percutaneous external drainage, the existence of liver fibrosis should be considered, which indicates a poor prognosis after the definitive operation; in such cases, further liver transplantation should be performed [34]. In our study, Case 1 presented symptoms in the infantile period, had already developed liver fibrosis on admission with a long-time biliary obstruction, and ultimately underwent liver transplantation. Therefore, US-guided percutaneous external drainage can help identify such cases. Similar to previous studies, early surgical intervention was recommended for all patients once a choledochal cyst was identified [1, 8, 12, 28, 34].

Many studies have shown that minimally invasive operations, including laparoscopic- or robot-assisted, have obvious advantages in treating choledochal cysts [1, 35, 36]. For complicated choledochal cyst, compared with traditional open external drainage, US-guided percutaneous external drainage can ensure minimally invasive treatment of the entire process. The giant choledochal cyst was conventionally treated by laparotomy, but in our study, except for Case 2, all subsequent patients underwent minimally invasive operations [18, 20]. This suggests that minimally invasive operation after US-guided percutaneous external drainage is safe and feasible for complicated choledochal cyst.

This study was limited by the small sample of patients, the isolated use of ultrasound, and the lack of a control group. Based on the present findings and the absence of a control group, it is not possible to prove the superiority of US-guided percutaneous external drainage over simple conservative treatments for patients with complicated choledochal cyst. Therefore, to what extent complicated choledochal cyst should be performed US-guided percutaneous external drainage before the definitive operation needs further studies. Further accumulation of cases is expected in the future.

## Conclusions

Our detailed analysis of this small cohort suggests that US-guided percutaneous external drainage is technically feasible for choledochal cyst with giant cysts or coagulopathy in children, which may provide suitable conditions for subsequent definitive operation and has a good prognosis.

## Data Availability

All data generated or analysed during this study are included in this published article.

## Abbreviations

US: ultrasound
CT: computed tomography
MRCP: magnetic resonance cholangiopancreatography
M: male
F: female
PBD: percutaneous biliary drainage
PTCD: percutaneous transhepatic cholangio-drainage
PTGD: percutaneous transhepatic gallbladder drainage
CRP: C-reactive protein
WBC: white blood cell
FIB: fibrinogen
PT: prothrombin time
APTT: activated partial thromboplastin time
NH_3_: ammonia
AST: aspartate aminotransferase
ALT: alanine aminotransferase
γ-GGT: γ-glutamyl transpeptidase
DBIL: direct bilirubin
IBIL: indirect bilirubin
AMY: amylase
LPS: lipase
CBD: common bile duct
